# Markers of bone turnover for the management of patients with bone metastases from prostate cancer

**DOI:** 10.1054/bjoc.1999.1012

**Published:** 2000-02

**Authors:** P Garnero, N Buchs, J Zekri, R Rizzoli, R E Coleman, P D Delmas

**Affiliations:** INSERM Research Unit 403, Hôpital E Herriot, Pav F, Lyon cedex 03, 69437, France; SYNARC, Lyon, France; Division of Bone Disease, University Hospital, Geneva, Switzerland; YCR Department of Clinical Oncology, Sheffield, UK

**Keywords:** bone markers, prostate cancer, bone metastases, type I collagen, bisphosphonate

## Abstract

Although increased bone formation is a prominent feature of patients with osteosclerotic metastases from prostate cancer, there is also some evidence for increased bone resorption. The aim of this study was to compare the clinical utility of new bone resorption markers to that of bone formation in patients with bone metastases from prostate cancer before and after bisphosphonate treatment. Thirty-nine patients with prostate cancer and bone metastasis, nine patients with prostate cancer without bone metastases, nine patients with benign prostatic hyperplasia and 355 healthy age-matched men were included. Urinary non-isomerized (α CTX) and β isomerized (β CTX) type I collagen C-telopeptides (CTX) and a new assay for serum CTX were used to assess bone resorption. Bone formation was determined by serum osteocalcin, serum total (T-ALP) and bone (BAP) alkaline phosphatase and serum type I collagen C-terminal propeptide (PICP). Fourteen patients with bone metastases were also evaluated 15 days after a single injection of the bisphosphonate pamidronate (120 mg). Levels of all bone formation and bone resorption markers were significantly (*P*< 0.006–0.0001) higher in patients with prostate cancer and bone metastasis than in patients with benign prostatic hyperplasia, patients with prostate cancer without bone metastases and healthy controls. In patients with bone metastases the median was increased by 67% for serum osteocalcin, 128% for T-ALP, 138% for BAP, 79% for PICP, 220% for urinary α CTX, 149% for urinary β CTX and 214% for serum CTX. After bisphosphonate treatment all three resorption markers significantly decreased by an average of 65% (*P* = 0.001), 71% (*P* = 0.0010) and 61% (*P* = 0.0015) for urinary α CTX, urinary β CTX and serum CTX, respectively, whereas no significant change was observed for any bone formation markers. Patients with prostate cancer and bone metastases exhibit a marked increase in bone resorption, which decreases within a few days of treatment with pamidronate. These findings suggest that these new resorption markers may be useful for the management of these patients. © 2000 Cancer Research Campaign


*British Journal of Cancer* (2000) **82**(4), 858–864
© 2000 Cancer Research Campaign
DOI: 10.1054/ bjoc.1999.1012, available online at http://www.idealibrary.com on IDEAL®

### Markers of bone turnover for the management of patients with bone metastases from prostate cancer

**P Garnero[1,2], N Buchs[3], J Zekri[4], R Rizzoli[3], RE Coleman[4] and PD Delmas[1]**

[1]INSERM Research Unit 403, Hôpital E Herriot, Pav F, 69437 Lyon cedex 03, France; [2]SYNARC, Lyon, France; [3]Division of Bone Disease, University Hospital, Geneva, Switzerland; [4]YCR Department of Clinical Oncology, Sheffield, UK

**Summary** Although increased bone formation is a prominent feature of patients with osteosclerotic metastases from prostate cancer, there is also some evidence for increased bone resorption. The aim of this study was to compare the clinical utility of new bone resorption markers to that of bone formation in patients with bone metastases from prostate cancer before and after bisphosphonate treatment. Thirty-nine patients with prostate cancer and bone metastasis, nine patients with prostate cancer without bone metastases, nine patients with benign prostatic hyperplasia and 355 healthy age-matched men were included. Urinary non-isomerized (α CTX) and β isomerized (β CTX) type I collagen C-telopeptides (CTX) and a new assay for serum CTX were used to assess bone resorption. Bone formation was determined by serum osteocalcin, serum total (T-ALP) and bone (BAP) alkaline phosphatase and serum type I collagen C-terminal propeptide (PICP). Fourteen patients with bone metastases were also evaluated 15 days after a single injection of the bisphosphonate pamidronate (120 mg). Levels of all bone formation and bone resorption markers were significantly ($P$ < 0.006–0.0001) higher in patients with prostate cancer and bone metastasis than in patients with benign prostatic hyperplasia, patients with prostate cancer without bone metastases and healthy controls. In patients with bone metastases the median was increased by 67% for serum osteocalcin, 128% for T-ALP, 138% for BAP, 79% for PICP, 220% for urinary α CTX, 149% for urinary β CTX and 214% for serum CTX. After bisphosphonate treatment all three resorption markers significantly decreased by an average of 65% ($P$ = 0.001), 71% ($P$ = 0.0010) and 61% ($P$ = 0.0015) for urinary α CTX, urinary β CTX and serum CTX, respectively, whereas no significant change was observed for any bone formation markers. Patients with prostate cancer and bone metastases exhibit a marked increase in bone resorption, which decreases within a few days of treatment with pamidronate. These findings suggest that these new resorption markers may be useful for the management of these patients. © 2000 Cancer Research Campaign

**Keywords:** bone markers; prostate cancer; bone metastases; type I collagen; bisphosphonate

Prostate cancer is the most common malignancy in elderly men and is often complicated by osteosclerotic metastases. Bone scintigraphy is commonly used to assess the extent of bone metastases; its use is limited in monitoring of treatment efficacy because it is an expensive, time-consuming technique that does not reflect the rapid skeletal response to therapy (Coleman, 1998). The bone scan flare response following successful therapy also reduces the value of bone scanning in the early monitoring of treatment in prostate cancer (Pollen et al, 1984). The serum level of prostate-specific antigen is the most sensitive index of disease progression in most but not all patients, but does not reflect the effects of palliative treatments such as bisphosphonates for bone metastases in patients that escape from antihormonal therapy.

The marked increase of osteoblastic activity in patients with osteosclerotic metastases is reflected by increased levels of serum total and bone-specific alkaline phosphatase (Pecherstorfer et al, 1995; Lorente et al, 1996). There is, however, biochemical (Myamoto et al, 1994, Sano et al, 1994; Kylmala et al, 1995; Berruti et al, 1996; Ikeda et al, 1996; Takeuchi et al, 1996; Maeda et al, 1997; Nguyen-Pamart et al, 1997, Pelger et al, 1998) and histological (Urwin et al, 1985; Clarke et al, 1991) evidence of increased bone resorption in these patients even in the absence of overt osteolytic bone metastases. This increased bone resorption is of clinical relevance as it is the rationale for using bisphosphonates, a palliative treatment that has been shown to reduce bone pain in patients with progressive metastatic prostate cancer who no longer respond to hormonal therapy (Adami et al, 1985; Carey and Lippert, 1988; Clarke et al, 1992; Kylmala et al, 1993, Taube et al, 1994; Pelger et al, 1998).

In this study we assessed the level of bone resorption in patients with osteosclerotic metastases from prostate cancer before and after bisphosphonate treatment by using new sensitive and specific biochemical markers of bone resorption including a serum assay for type I collagen C-telopeptide breakdown products; and values were compared to those of bone formation markers.

*Received 22 March 1999*
*Revised 20 September 1999*
*Accepted 20 September 1999*

*Correspondence to:* P Garnero

### PATIENTS AND METHODS

Forty-eight patients with carcinoma of the prostate (age 71.7 ± 9.6 years, mean ± 1 s.d.) and nine with benign prostatic hyperplasia (BHP) (age 69.8 ± 4.2 years) were enrolled. All patients with cancer had a prostatic biopsy and tissue diagnosis of adenocarcinoma. Bone involvement and its extent was evaluated by bone scintigraphy using Technetium-99m labelled methylene bisphosphonate. When bone metastases were suspected, confirmation was obtained with standard radiographs, computerized tomography (CT) and/or magnetic resonance imaging. The metastatic load in bone, defined by the number and size of the metastatic lesions, was graded using the scoring and stratification proposed by Soloway et al (1988) in a subset of 32 patients with prostate cancer. Patients were classified into two groups: score < 2 (i.e. 0 and 1) corresponding to fewer than six malignant bone lesions; score ≥ 2 (from 2 to 4) corresponding to six and more lesions. Among the subjects with prostate cancer, 39 had proven bone metastases (age 71.8 ± 9.6 years), whereas nine (age 71.1 ± 9.8 years) had no detectable metastatic diseases. The nine patients with BPH had no previous history of malignancy and the biopsy specimen did not reveal any evidence of cancer. Forty-eight out of the 57 patients had no previous treatment for their prostate disease. Five subjects with skeletal metastases had undergone orchidectomy 4–56 months before the study (23 ± 4 months) and two had a previous hormonal therapy (cyproterone acetate for 3 months, flutamide for 8 months). Fourteen patients with endocrine-resistant bone metastases were also assessed 15 days after a single intravenous injection of the bisphosphonate pamidronate (120 mg). All these 14 patients had apparently sclerotic disease with no radiographical evidence of lytic lesions. None of the patients had renal insufficiency.

Bone marker levels in patients with prostate cancer and BPH were compared to those of 355 healthy age-matched men (mean age 70.1 ± 5.9 years: 51–85 years) taken from a large population-based cohort involved in a prospective study on the determinants of bone loss in men (MINOS study). None of the controls had a disease or treatment that may affect bone metabolism. These studies were approved by the local ethical committee and informed consent was obtained from all participants.

Fasting serum and 2-h urine samples were collected in all patients for measuring biochemical markers of bone turnover.

### Bone markers

Markers of bone formation included serum total osteocalcin (ELSA-OSTEO, Cis biointernational, Gif/Yvette, France) (Garnero et al, 1992), total alkaline phosphatase activity (T-ALP) measured by a colorimetric method (Alkaline phosphatase® AMP-buffer, Boehringer, Germany), serum bone-specific alkaline phosphatase (BAP, Alkaphase B, Metra Biosystems, Mountain View, CA, USA) (Gomez et al, 1995) and serum type I collagen C-terminal propeptide (PICP, Prolagen-C™, MetraBiosystems®, Palo Alto, CA, USA) (Winterbottom et al, 1993). Bone resorption was assessed by measuring the urinary excretion of non-isomerized (α) and β isomerized (β) C-telopeptides of type I collagen (CTX) by alpha Crosslaps RIA (Osteometer Biotech, Herlev, Denmark) and Crosslaps enzyme-linked immunosorbant assay (ELISA) (CIS Biointernational, Gif/Yvette, France) respectively (Bonde et al, 1994, 1996). We also measured the serum concentration of CTX using a new immunoassay (Serum Crosslaps one-step™, CIS Biointernational®, Gif/Yvette, France). This serum resorption marker assay uses two monoclonal antibodies raised against a synthetic peptide with an amino acid sequence specific for a part of the C-telopeptide of the α 1 chain of type I collagen (Glu-Lys-Ala-His-βAsp-Gly-Gly-Arg) (Bonde et al, 1997; Rosenquist et al, 1998). Intra- and interassay CVs are lower than 5 and 8% respectively and the sensitivity is 0.15 nmol/l.$^{-1}$

### Statistical analyses

Significance of the difference between groups was assessed using non-parametric tests (Mann–Whitney rank test or Kruskall–Wallis tests) where the α risk was corrected according to Bonferroni's procedure. Relationships between markers were assessed by Spearman (rank) correlation analysis. The effect of bisphosphonate treatment was assessed by the non-parametric Wilcoxon paired test.

### RESULTS

All bone turnover markers were significantly higher in patients with bone metastases from prostate cancer compared to healthy age-matched controls, localized prostate cancer and patients with BPH (Table 1). No significant difference was found between healthy men, patients with BPH and those with localized prostate cancer. For bone formation, T-ALP, BAP and PICP provided similar associations with bone metastases, whereas serum osteocalcin was less increased. Indeed, the sensitivity defined as the proportion of patients with bone metastases exhibiting bone marker levels higher than the mean + 2 standard deviations (s.d.) of normals was 62% for T-ALP, 62% for BAP, 60% for PICP and only 43% for osteocalcin (Figure 1). No patient with BPH or localized prostate cancer demonstrated increased T-ALP, BAP and PICP levels. The three resorption markers demonstrated a similar performance in the detection of bone metastases with a sensitivity of 61%, 53% and 64% for urinary α CTX, urinary β CTX and serum CTX respectively (Figure 1). In patients with bone metastases, we found significant correlation between all markers investigated with, however, lowest values with serum osteocalcin (Table 2). When patients with prostate cancer were divided in two groups according to the bone metastatic load, higher levels of all bone

**Table 1** Markers of bone turnover in controls and patients with prostate diseases. Results are shown as median (interquartile range)

| | Healthy controls ($n$ = 355) | BPH[a] $n$ = 9) | PC. M–[b] ($n$ = 9) | PC. M+[c] ($n$ = 39) | $P$[e] |
|---|---|---|---|---|---|
| Osteocalcin (ng ml$^{-1}$) | 17.8 (7.8) | 18.2 (9.8) | 16.5 (11.7) | 29.8[d] (28.6) | 0.005 |
| T-ALP (IU l$^{-1}$) | 50 (21) | 45 (18) | 38 (10) | 114[d] (192) | < 0.0001 |
| BAP (ng ml$^{-1}$) | 16.2 (6.8) | 13.5 (6.7) | 8.7 (3.6) | 38.5[d] (105) | < 0.0001 |
| PICP (ng ml$^{-1}$) | 94 (33) | 74 (33) | 61 (33) | 168 (121) | < 0.0001 |
| Urinary α CTX (μg mmol$^{-1}$ Cr) | 194 (140) | 197 (253) | 184 (430) | 621[d] (1165) | 0.0056 |
| Urinary β CTX (μg mmol$^{-1}$ Cr) | 146 (90) | 120 (188) | 89 (214) | 363[d] (650) | 0.0056 |
| Serum CTX (nmol l$^{-1}$) | 2.2 (1.3) | 2.7 (2.3) | 2.5 (3.5) | 6.9[d] (8.4) | 0.0008 |

[a]Benign prostate hyperplasia patients; [b]patients with prostate cancer without bone metastases; [c]patients with prostate cancer with bone metastases. No significant difference for any marker between healthy controls, BPH and PC. M–. [d] $P$ < 0.0001 vs healthy controls. [e]Comparison between PC. M + and BPH+PC. M–.

© 2000 Cancer Research Campaign

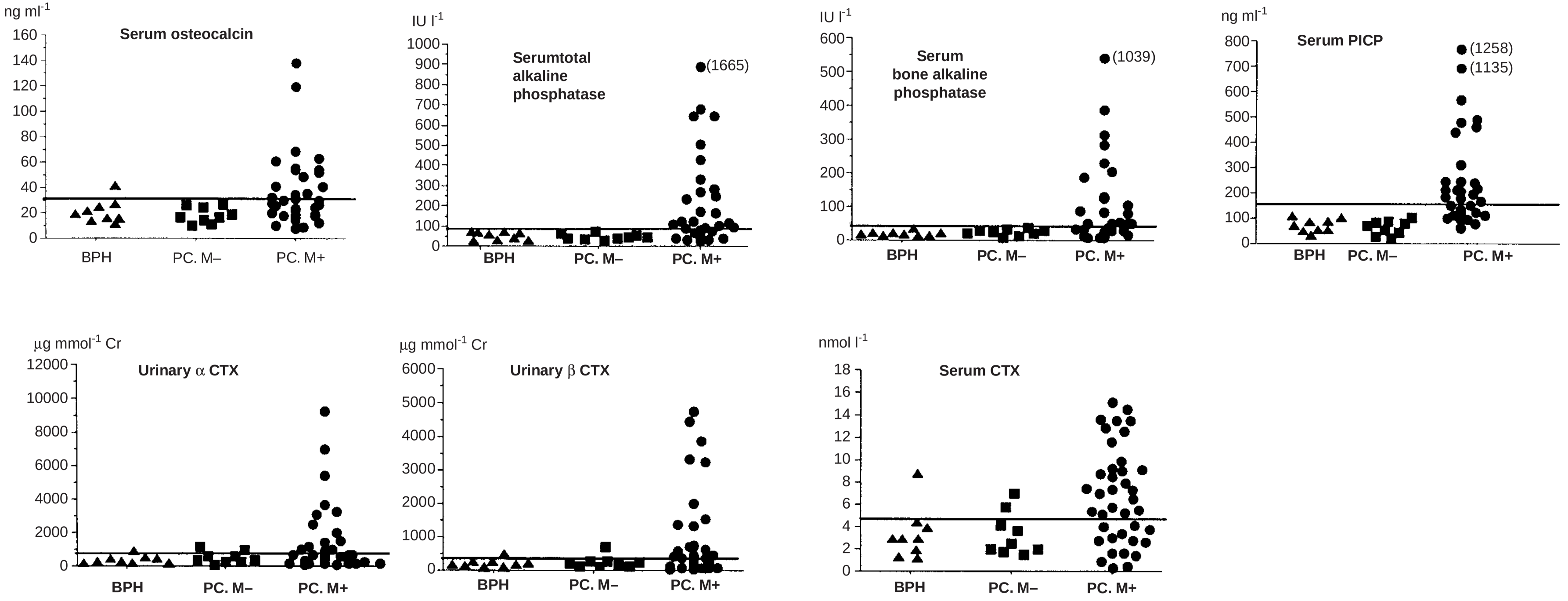

**Figure 1** Individual levels of four bone formation (osteocalcin, total and bone alkaline phosphatase and PICP) and three bone resorption (urinary α CTX, urinary β CTX, serum CTX) markers in patients with benign prostatic hypertrophy (BPH), patients with prostate cancer without bone metastases (PC. M–) and patients with prostate cancer with bone metastases (PC. M+). The horizontal line represents the upper limit of the normal range (mean + 2 s.d.) established in 355 healthy age-matched men

© 2000 Cancer Research Campaign

**Table 2** Correction between biochemical markers of bone turnover in patients with bone metastases from prostate cancer

| | **Osteocalcin** | **T-ALP** | **BAP** | **PICP** | **Urinary α CTX** | **Urinary β CTX** |
|---|---|---|---|---|---|---|
| T-ALP | 0.43 (0.01) | | | | | |
| BAP | 0.36 (0.03) | 0.98 (<0.0001) | | | | |
| PICP | 0.46 (0.007) | 0.79 (< 0.0001) | 0.82 (< 0.0001) | | | |
| Urinary α CTX | 0.46 (0.005) | 0.56 (0.0012) | 0.59 (0.0003) | 0.57 (0.0001) | | |
| Urinary β CTX | 0.45 (0.007) | 0.52 (0.003) | 0.51 (0.002) | 0.51 (0.004) | 0.94 (< 0.0001) | |
| Serum CTX | 0.38 (0.02) | 0.65 (0.0002) | 0.66 (< 0.0001) | 0.64 (0.0002) | 0.73 (< 0.0001) | 0.67 (< 0.0001) |

Results are shown as the Spearman correlation coefficient (significance level).

**Table 3** Bone turnover and bone metastatic load in patients with prostate cancer

| | **Soloway score** | | |
|---|---|---|---|
| | **< 2** *n* = 14 | **≥ 2** *n* = 18 | ***P*** |
| Osteocalcin (ng ml$^{-1}$) | 20.7 (9.9) | 28.5 (36.3) | 0.03 |
| T-ALP (IU l$^{-1}$) | 41 (21) | 108 (196) | 0.0003 |
| BAP (ng ml$^{-1}$) | 9.3 (6.0) | 40.6 (114) | <0.0001 |
| PICP (ng ml$^{-1}$) | 70 (44) | 182 (191) | 0.0004 |
| Urinary α CTX (μg mmol$^{-1}$ Cr) | 222 (477) | 467 (1324) | 0.11 |
| Urinary β CTX (μg mmol$^{-1}$ Cr) | 101 (258) | 194 (549) | 0.26 |
| Serum CTX (nmol l$^{-1}$) | 3.9 (3.8) | 8.4 (10.8) | 0.01 |

The Soloway score was assessed by isotopic bone scintigaphy. Score < 2 was defined by fewer than 6 bone metastases and score ≥ 2 by 6 or more bone metastases. Results are shown as median (interquartile range).

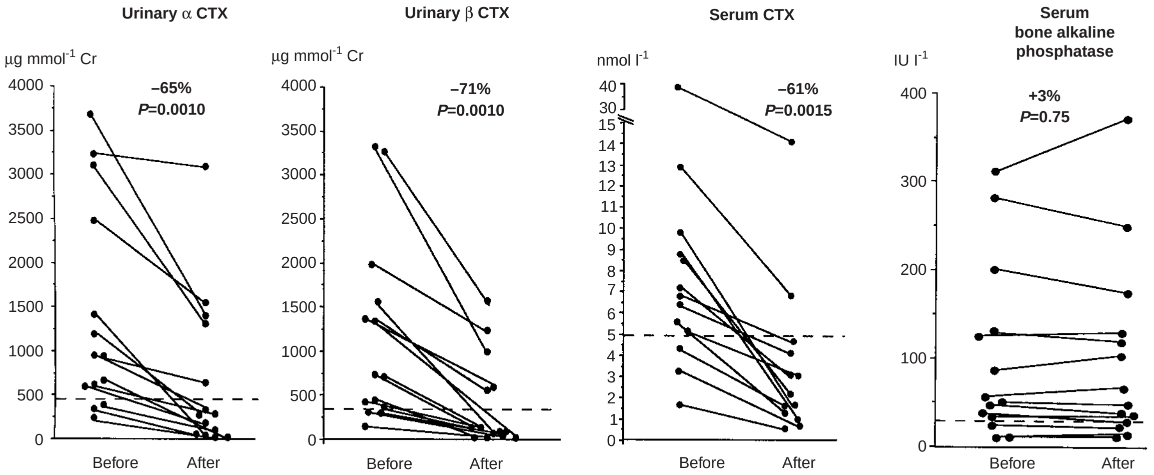

**Figure 2** Response of 3 bone resorption markers and serum bone alkaline phosphatase to bisphosphonate treatment in patients with prostate cancer with bone metastases. Levels of each marker are shown before treatment and 15 days after a single i.v. of pamidronate (120 mg). The dotted line represents the upper limit of the normal range as defined in Figure 1

formation and bone resorption markers were observed in those with a Soloway score ≥ 2 compared to those with a score < 2, with however no significant difference for the two urinary resorption markers (Table 3).

The effect of bisphosphonate treatment was assessed in a subset of the patients with bone metastases. A single injection of pamidronate induced a decrease of the three resorption markers in all patients, with a mean fall of 60–70%. Before treatment, 70–80% of patients had levels above the upper limit of the normal range, whereas only 15–35% had increased levels 15 days after the injection of bisphosphonate (Figure 2). No significant change in osteocalcin (–8 ± 18%, *P* = 0.35); T-ALP (–2 ± 23%, *P* = 0.73), BAP (+ 3 ± 21%, *P* = 0.75) or PICP (–12 ± 28%, *P* = 0.20) was observed after 15 days of treatment (Figure 2).

*© 2000 Cancer Research Campaign*

### DISCUSSION

In this study, we found that patients with bone metastases from prostate cancer are characterized by an increased bone formation and by a marked increase in bone resorption as assessed by several biochemical markers of bone resorption, including a new assay for serum type I collagen fragments. Markers of bone resorption, but not those of bone formation, were significantly reduced within 15 days after a treatment with bisphosphonate.

Amongst the bone formation markers, our study confirms the higher association of serum T-ALP, BAP and PICP over serum osteocalcin with bone metastases (Koizumi et al, 1997; Nakashima et al, 1997; Yoshida et al, 1997). Of interest, T-ALP, which is still the parameter most widely used for assessing bone metastases, was as sensitive as BAP, suggesting that this non-specific bone turnover marker is a valuable index of bone formation in this condition. However, others reported a higher diagnostic value of BAP compared to T-ALP especially for stratifying the degree of bone metastases (Akimoto et al, 1998). The reasons for the lower sensitivity of serum osteocalcin are unclear, but this situation is reminiscent of that found in Paget's disease of bone (Delmas et al, 1986), which, like prostate cancer, is characterized by a marked increase of bone turnover with the formation of newly woven bone. After secretion by the osteoblastic cells, part of the newly synthesized osteocalcin is captured by bone matrix and part is released in the circulation. In Paget's disease, the low sensitivity of serum osteocalcin has been suggested to be related to an increased uptake of newly synthesized osteocalcin by the woven bone, resulting in a decrease of the fraction released into the circulation. This may also be related to the involvement of osteoblasts at different stages of differentiation. Indeed, PICP is believed to be a marker of the early or proliferation phase, BAP a marker of matrix maturation phase and osteocalcin a marker of late bone formation.

Most of the resorption marker assays are currently using urine samples. Urinary resorption markers have several limitations, including the need for control of sampling time and correction for urinary creatinine, and a large within patient variability (Garnero et al, 1994; Hannon et al, 1998). Urinary collection is, moreover, often cumbersome in clinical practice. A serum-based assay measuring carboxyterminal type I collagen telopeptide (ICTP) has been developed (Risteli et al, 1993) and increased ICTP levels have been reported in prostate cancer patients with bone metastases (Kylmala et al, 1995; Yoshida et al, 1997; Akimoto et al, 1998). However, the tissue specificity and the clinical significance of serum ICTP levels are still unclear. In particular, ICTP levels increase after treatment with anabolic steroids, which are believed to decrease bone resorption and to stimulate collagen synthesis (Hassager et al, 1994). Thus, ICTP appears to be more a marker of collagen turnover than of bone resorption.

In the present study we used a new serum bone resorption marker that has been shown to specifically reflect bone resorption (Bonde et al, 1997; Rosenquist et al, 1998). This is the first study demonstrating its utility for monitoring osteosclerotic metastases from prostate cancer. The sensitivity of serum CTX to detect bone metastases compares very well with that of urinary measurements of α and β CTX and with those previously reported for the conventional, but cumbersome high performance liquid chromatography measurement of total excretion of pyridinoline and deoxypyridinoline (Myamoto et al, 1994; Sano et al, 1994; Takeuchi et al, 1996). Interestingly, serum CTX levels correlated with the metastatic load in bone as assessed by the Soloway score in contrast to the two urinary resorption markers. These data suggest that this new serum CTX assay represents an adequate alternative to the urinary resorption markers for assessing bone involvement in prostate cancer. Serum CTX appears to reflect more accurately the extent of bone disease, probably due to the lower precision error of serum compared to urinary measurements (Christgau et al, 1997). Nevertheless, because the sensitivity for all markers investigated ranged from 53 to 64%, biochemical markers are likely to be of little help in the diagnosis of secondary bone involvement in prostate cancer.

Bisphosphonates, which are potent inhibitors of bone resorption, are an important adjuvant treatment for the management of metastatic bone disease. Bisphosphonates are the treatment of choice for hypercalcaemia of malignancy (Body et al, 1996) and have been shown to reduce skeletal complications in multiple myeloma (Berenson et al, 1996) and breast cancer (Hortobayi et al, 1996). They relieve metastatic bone pain caused by a variety of solid tumours, including prostate, with a consequent improvement in quality of life (Adami et al, 1985; Carey and Lippert, 1988; Coleman et al, 1996; Pelger et al, 1998). Interestingly, it has been suggested that the pretreatment levels, the magnitude of the change of the decreases under treatment and the levels reached after treatment of some urinary resorption markers are predictive of the efficacy of pamidronate in reducing pain in normocalcaemic carcinoma patients (Vinholes et al, 1996, 1997; Adami et al, 1997), suggesting that these markers may be useful to monitor the efficacy of bisphosphonate treatment in patients with bone metastases.

One of the main criteria to judge the clinical utility of bone markers is the time-course and magnitude of the response under treatment. Fifteen days after treatment with bisphosphonate none of the four markers of bone formation decreased contrasting with the significant reduction of the bone resorption markers. Because the renal clearance of osteocalcin and the hepatic clearance of BAP and PICP are very rapid with a half-life lower than 6 min for all three markers (Price et al, 1981; Young et al, 1984; Smedsrod et al, 1990; Blom et al, 1998), if pamidronate has decreased the release of these markers from bone to blood, a significant decrease in their circulating levels would also has been observed after 15 days. More likely, the absence of decrease of bone formation markers results from the direct inhibitory action of the aminobisphosphonate pamidronate on bone resorption, followed by an indirect decrease in bone formation through the operation of the coupling mechanism. This short-term response is similar to that previously found in patients with Paget's disease of bone treated by pamidronate (Uebelhart et al, 1990) and more recently in patients with prostate cancer treated with another bisphosphonate, olpadronate (Pelger et al, 1998).

We observed a 60–70% decrease of the levels of bone resorption markers including serum CTX after treatment with bisphosphonate, i.e. a magnitude similar to that reported by Vinholes et al (1997) for urinary NTX using a similar treatment protocol. It should be pointed out, however, that in a large proportion of patents with high levels to start with, the biochemical markers of bone resorption remained increased after treatment. Similar results have been observed when bone resorption was assessed by urinary hydroxyproline and this was ascribed to the contribution of metastatic involvement of soft tissues to the excess of hydroxyproline excretion. The inability of a single dose of pamidronate to

 © 2000 Cancer Research Campaign normalize levels of three bone-specific resorption markers suggest that more intensive schedules, higher doses or more potent bisphosphonates will be required to normalize bone turnover in most patients.

The three different biochemical markers of bone resorption used in this study recognize fragments of the C-telopeptide of the alpha 1 chain of type I collagen and all of them used antibodies raised against an amino acid sequence containing a lysine involved in the cross-linking of collagen molecules. Although, α CTX and β CTX assays could at least theoretically detect degradation products from newly-synthesized molecules that have not participated in fibre formation, it has been shown that the majority of urinary fragments detected by these two assays are linked by pyridinoline, deoxypyridinoline or a non-fluorescent cross-link (Fledelius et al, 1997). Since the two monoclonal antibodies used in the serum CTX assay must bind to the same antigen simultaneously for detection to occur, it is a prerequisite that the type I collagen fragments measured are cross-linked (Rosenquist et al, 1998). Thus, all three assays are likely to reflect mainly the degradation of type I collagen molecules, which have participated in fibre formation.

However, the different assays differ by their ability to detect the β isomerization process which is a post-translational modification believed to be associated with ageing of proteins. Indeed, the urinary α CTX assay recognizes non-isomerized fragments, whereas urinary β CTX assay and serum CTX recognize β isomerized fragments and thus are likely to reflect degradation of aged bone matrix. We previously found that in patients with Paget's disease of bone, the increase of urinary α CTX was much more important than that of β CTX because the woven bone characterizing these patients is comprised mainly of non-isomerized type I collagen molecules as indicated by immunohistochemistry (Garnero et al, 1997). Because patients with bone metastases from prostate cancer, like patients with Paget's disease, are characterized by a marked increase of bone turnover in localized area of the skeleton, we could expect a higher increase of α CTX compared to β CTX. Such a hypothesis has been raised recently by Engler et al (1998) to explain the better sensitivity of serum ICTP – a marker believed to detect both non-isomerized and β isomerized fragments – compared to urinary β CTX for detecting osteolytic metastases and the difference in their response after treatment with pamidronate. In contrast in our study, we found similar increases over controls and response to treatment of urinary α CTX, urinary β CTX and serum CTX, suggesting that in our cohort of prostate cancer patients the pattern of β isomerization of type I collagen was not altered. There are several reasons that could explain these differences. In the study of Engler et al (1998) most of the patients had bone metastases from breast involvement and only 19 from prostate cancer. They observed the effect of bisphosphonate treatment after 28 days compared to 15 days in our study. According to our data in patients with Paget's disease of bone, short-term treatment with bisphosphonate induces similar decreases of non-isomerized and β isomerized CTX, whereas with advancing time differences between the two markers occur (Garnero et al, 1998). In addition as pointed out before, because ICTP is not a specific marker of bone resorption, its large increase in untreated cancer patients and the absence of significant decrease under bisphosphonate observed by Engler et al (1998) could also result in part from the contribution of soft tissues to serum ICTP levels.

Our study has some limitations. Patients were not separated according to the type of metastases, i.e. purely sclerotic or mixed. Thus, we could not investigate whether or not markers reflecting the degradation of non-isomerized and β isomerized CTX could give different information in these two groups; further studies in this area are required. We did not investigate the value of biochemical markers to predict the efficacy of bisphosphonate to reduce bone pain – a relationship that we previously reported for other markers (Vinholes et al, 1996, 1997) – although just 2-week time points is probably too few to adequately address this issue.

In conclusion, using a panel of recently developed markers of bone resorption, including a new serum assay for type I collagen breakdown products, we found that patients with prostate cancer are characterized by a marked increase of bone resorption which decreases within 2 weeks of treatment with high dose pamidronate, whereas no significant change was observed for markers of bone formation. These data suggest that new assays for urinary and serum CTX may be useful to monitor its efficacy.

### REFERENCES

Adami S (1997) Bisphosphonate in prostate cancer. *Cancer* **80:** 1674–1679

Adami S, Salvagno G, Guarrera, Bianchi G, Dorizzi R and Rosini S (1985) Dichloromethylene diphosphonate in patients with prostatic carcinoma metastatic to the skeleton. *J Urol* **134**: 1152–1154

Akimoto S, Furuya Y, Akakura K and Ito H (1998) Comparison of markers of bone formation and resorption in prostate cancer patients to predict bone metastases. *Endocrine J* **45**: 97–104

Berenson JR, Lichtensein A, Poter L, Di MoPoulos MA, Bordini C, George S, Lipton A, Keller A, Ballester O, Koracs MJV, Blacklock HA, Bell R, Simeone J, Reitsma DJ, Heffernan PD, Seaman J and Knight RD (1996) Efficacy of pamidronate in reducing skeletal events in patients with advanced myeloma. *N Engl J Med* **334**: 488–493

Berruti A, Piovesan A, Torta M, Raucci CA, Gorzegno G, Paccoti P, Doglotti L and Angeli A (1996) Biochemical evaluation of bone turnover in cancer patients: relationship with radiograph appearances and disease extension. *Br J Cancer* **73**: 1581–1587

Blom E, Ali MM, Mortensen B and Huseby NE (1998) Elimination of alkaline phosphatases from circulation by the galactose receptor. Different isoforms are cleared at various rates. *Clin Chim Acta* **270**: 125–137

Body J-J, Coleman RE and Piccart M (1996) Use of bisphosphonates in cancer patients. *Cancer Treat Rev* **22**: 265–287

Bonde M, Qvist P, Fledelius C, Riis BJ and Christiansen C (1994) Immunoassay for quantifying type I collagen degradation products in urine evaluated. *Clin Chem* **40**: 2022–2025

Bonde M, Fledelius C, Qvist P and Christiansen C (1996) A coated-tube radioimmunoassay for C-telopeptides of type I collagen to assess bone resorption. *Clin Chem* **42**: 1639–1644

Bonde M, Garnero P, Fledelius C, Qvist P, Delmas PD and Christiansen C (1997) Measurement of bone degradation products in serum using antibodies reactive with an isomerized form of an eight amino acid sequence of the C-telopeptide of type I collagen. *J Bone Miner Res* **12**: 1028–1034

Carey PO and Lippert MC (1988) The treatment of painful prostatic bone metastases with oral etidronate sodium. *Urology* **32**: 403–407

Christgau S, Alexendersen P, Schlemmer A, Bonde M and Christiansen C (1997) Biological variation of the serum concentration of degradation products derived from the C-terminal telopeptide of type I collagen measured by a new version of the Crosslaps ELISA. *J Bone Miner Res* **12**: S497

Clarke NW, McClure J and George NJR (1991) Morphometric evidence for bone resorption and replacement in prostate cancer. *Brit J Urol* **68**: 74–80

Clarke NW, McClure J and George NJR (1992) Dissodium pamidronate identifies differential bone resorption in metastatic prostate cancer. *Br J Urol* **69**: 64–70

Coleman RE (1998) Monitoring of bone metastases. *Eur J Cancer* **34**: 252–259.

Coleman RE, Purohit OP and Vinholes J (1996) Improved quality of life with high dose bisphosphonate treatment: The UK experience. In: *Bisphosphonates – Improved Treatment of Osteolysis in Malignant and Non-malignant Indications*, Possinger K and Ziegler R (eds), pp. 37–48. Excerpta Oncologica Ciba Wehr/Baden, Ciba Geigy Verlag

Delmas PD, Demiaux B, Malaval L, Chapuy MC and Meunier PJ (1986) Serum bone-GLA-protein is not a sensitive marker of bone turnover in Paget's disease of bone. *Calcif Tissue Int* **38**: 60–61

© 2000 Cancer Research Campaign

Elomaa I, Kylmala T and Tammela T (1992) Effect of oral clodronate on bone pain. A controlled study in patients with metastatic prostate cancer. *Int Urol Nephrol*, **24**: 159–166
Engler H, Koeberle D, Thuerlimann B, Senn HJ and Riesen WF (1998) Diagnostic and prognostic value of biochemical markers in malignant bone disease: a prospective study on the effect of bisphosphonate on pain intensity and progression of malignant bone disease. *Clin Chem Lab Med* **36**: 879–885
Fledelius C, Johnsen AH, Cloos PAC, Bonde M and Qvist P (1997) Characterization of urinary degradation products derived from Type I collagen. *J Biol Chem* **272**: 9755–9763
Garnero P, Grimaux M, Demiaux B, Preaudat C, Seguin P and Delmas PD (1992) Measurement of serum osteocalcin with a human-specific two-site immunoradiometric assay. *J Bone Miner Res* **7**: 1389–1398
Garnero P, Shih WJ, Gineyts E, Karpf D and Delmas PD (1994) Comparison of new biochemical markers of bone turnover in late postmenopausal osteoporotic women in response to alendronate. *J Clin Endocrinol Metab* **79**: 1693–1700
Garnero P, Fledelius C, Gineyts E, Serre C-M, Vignot E and Delmas PD (1997) Decreased β isomerisation of C-telopeptides of type I collagen in Paget's disease of bone. *J Bone Miner Res* **12**: 1407–1415
Garnero P, Gineyts E, Shaffer AV, Seaman S and Delmas PD (1998) Measurement of urinary excretion of nonisomerized and β-isomerized forms of type I collagen breakdown products to monitor the effects of the bisphosphonate zoledronate in Paget's disease. *Arthritis Rheum* **41**: 354–360
Gomez B, Ardakani S, Ju J, Jenkins D, Cerelli M-J, Daniloff GY and Kung VT (1995) Monoclonal antibody assay for measuring bone-specific alkaline phosphatase in serum. *Clin Chem* **41**: 1560–1566
Hannon R, Blumsohn A, Naylor K and Eastell R (1998) Response of biochemical markers of bone turnover to hormone replacement therapy: impact of biological variability. *J Bone Miner Res* **13**: 1124–1133
Hassager C, Jensen LT, Podenphant J, Thomsen K and Christiansen C (1994) The carboxy-terminal pyridinoline cross-linked telopeptide of type I collagen in serum as a marker of bone resorption: the effect of Nandrolone decanoate and hormone replacement therapy. *Calcif Tissue Int* **54**: 30–33
Hortobagyi GN, Theriault RL, Porter L, Blayney D, Lipton A, Sinoff C, Wheller H, Simeone JF, Seaman J, Knight RD, Heffernan M and Reitsman DJ (1996) Efficacy of pamidronate in reducing skeletal events in patients with breast cancer and lytic bone metastases. *N Engl J Med* **335**: 1785–1791
Ikeda I, Miura T and Kondo I (1996) Pyridinium crosslinks as urinary markers of bone metastases in patients with prostate cancer. *Br J Urol* **77**: 102–106
Koizumi M, Maeda H, Yoshimura K, Yamauchi T, Kawai T and Ogata E (1997) Dissociation of bone metabolic markers in bone metastasis of prostate cancer. *Br J Cancer* **75**: 1601–1604
Kylmala T, Tammela T, Risteli L, Risteli J, Taube T and Elomaa I (1993) Evaluation of the effect of oral clodronate on skeletal metastases with type I collagen metabolites. A controlled trial of the Finnish Prostate Cancer Group. *Eur J Cancer* **29A**: 821–825
Kylmala T, Risteli J and Elomaa I (1995) Type I collagen degradation product (ICTP) gives information about the nature of bone metastases and has prognostic value in prostate cancer. *Br J Cancer* **71**: 1061–1064
Lorente JA, Morote J, Raventos C, Encabo G and Valenzuela H (1996) Clinical efficacy of bone alkaline phosphatase and prostate specific antigen in the diagnosis of bone metastasis in prostate cancer. *J Urol* **155**: 1348–1351
Maeda H, Koizumi M, Yoshimura K, Yamauchi T, Kawai T and Ogata E (1997) Correlation between bone metabolic markers and bone scan in prostatic cancer. *J Urol* **157**: 539–543
Myamoto KK, McSkerry SA, Robins SA, Besterman J and Mohler JL (1994) Collagen cross-link metabolites in urine as markers of bone metastases in prostatic carcinoma. *J Urol* **151**: 909–913
Nakashima J, Sumitomo M, Miyajima A, Jitsukawa S, Saito S, Tachibana M and Murai M (1997) The value of serum carboxyterminal propeptide of type I procollagen in predicting bone metastases in prostate cancer. *J Urol* **157**: 1736–1739
Nguyen-Pamart M, Caty A, Feutrie ML, Fournier C, Gosselin P and Mazeman E (1997) The diagnostic value of urinary Crosslaps and serum alkaline phosphatase in patients with prostate cancer. *Br J Urol* **80**: 452–455
Pecherstorfer M, Zimmer-Roth J, Shilling T, Woitge HW, Schmidt H, Baumgartner G, Thiebaud D, Ludwig H and Seibel MJ (1995) The diagnostic value of urinary pyridinium crosslinks of collagen, alkaline phosphatase and urinary calcium in neoplastic bone disease. *J Clin Endocrinol Metab* **80**: 97–103
Pelger RCM, Hamdy NAT, Zwinderman AH, Lycklama AAB, Nijeholt A and Papapoulos SE (1998) Effects of bisphosphonate olpadronate in patients with carcinoma of the prostate metastatic to the skeleton. *Bone* **22**: 403–408
Pollen JJ, Witztun KF and Ashburn WL (1984) The flare phenomenon on radionuclide bone scan in metastatic prostate cancer. *Am J Radiother* **142**: 773–776
Price PA, Williamson MK and Lothinger JW (1981) Origin of the vitamin K-dependent bone protein found in plasma and its clearance by kidney and bone. *J Biol Chem* **256**: 12760–12766
Risteli J, Elomaa I, Niemi S, Novamo A and Risteli L (1993) Radioimmunoassay for the pyridinoline cross-linked carboxy-terminal telopeptide of type I collagen: a new serum marker of bone collagen degradation. *Clin Chem* **39**: 635–640
Rosenquist C, Fledelius C, Christgau S, Pedersen BJ, Bonde M, Qvist P and Christiansen C (1998) Serum crosslaps one-step ELISA. First application of monoclonal antibodies for measurement in serum of bone-related degradation products from C-terminal telopeptides of type I collagen. *Clin Chem* **44**: 2281–2289
Sano M, Kushida K, Takahashi M, Ohishi T, Kawana M, Okada M and Inoue T (1994) Urinary pyridinoline and deoxypyridinoline in prostate carcinoma patients with bone metastases. *Br J Cancer* **70**: 701–703
Smedsrod B, Melkko J, Risteli L and Risteli J (1990) Circulating C-terminal propeptide of type I procollagen is cleared mainly via the mannose receptor in liver endothelial cells. *Biochem J* **271**: 345–350
Soloway MS, Hardeman SW, Hickey D, Raymond JT, Todd B and Soloway S (1988) Stratification of patients with metastatic prostate cancer based on extent of disease on initial bone scan. *Cancer* **61**: 195–202
Takeuchi S-I, Arai K, Saitoh H, Yoshida K-I and Miura M (1996) Urinary pyridinoline and deoxypyridinoline as potential markers of bone metastases in patients with prostate cancer. *J Urol* **156**: 1691–1695
Taube T, Kylmälä TC, Lamberq-Allard C, Tammela TLJ and Elomaa I (1994) The effect of clodronate in metastatic prostate cancer: histomorphometric report of a double-blind randomised placebo controlled study. *Eur J Cancer* **30**: 751–758
Uebelhart D, Gineyts E, Chapuy MC and Delmas PD (1990) Urinary excretion of pyridinium cross links: a new marker of bone resorption in metabolic bone disease. *Bone Miner* **8**: 87–96
Urwin GH, Percival RC, Harris S, Beneton MNC, Williams JL and Kanis SA (1985) Generalised increase in bone resorption in carcinoma of prostate. *Eur J Urol* **57**: 721–723
Vinholes JJ, Guo C-Y, Purohit OP, Eastell R and Coleman RE (1996) Metabolic effects of pamidronate in patients with metastatic bone disease. *Br J Cancer* **73**: 1089–1095
Vinholes JJ, Purohit OP, Abbey ME, Eastell R and Coleman RE (1997) Relationships between biochemical and symptomatic response in a double-blind trail of pamidronate for metastatic disease. *Ann Oncol* **8**: 1243–1250
Winterbottom N, Vernon S, Freeman K, Daniloff G and Seyedin S (1993) A serum immunoassay for the C-terminal propeptide of type I collagen. *J Bone Miner Res* **8**: S341.
Young GP, Rose IS, Cropper S, Seetharam S and Alpers DH (1984) Hepatic clearance of rat intestinal alkaline phosphatase. *Am J Physiol* **274**: G419–426.
Yoshida K-I, Sumi S, Arai K, Koga F, Umeda H, Hosoya Y, Honda M, Yano M, Moriguchi H and Kitahara S (1997) Serum concentration of type I collagen metabolites as a quantitative marker of bone metastases in patients with prostate cancer. *Cancer* **80**: 1760–1767

 *© 2000 Cancer Research Campaign*
